# Risk stratification for intracranial infection after high-grade gliomas surgery: a nomogram development and validation study

**DOI:** 10.3389/fonc.2025.1697966

**Published:** 2025-11-19

**Authors:** Shichao Liu, Risheng Liang

**Affiliations:** Department of Neurosurgery, Fujian Medical University Union Hospital, Fuzhou, Fujian, China

**Keywords:** intracranial infection, high-grade gliomas surgery, risk factors, nomogram, predict

## Abstract

**Background:**

Intracranial infection (ICI) is a severe complication following high-grade gliomas (HGGs) surgery, leading to increased morbidity and mortality. This study aimed to identify risk factors for postoperative ICI and to develop and validate a nomogram for predicting its occurrence, providing a tool to guide clinical prevention.

**Methods:**

The clinical data of 104 patients who underwent surgery for HGGs between January 2017 and August 2024 were retrospectively analyzed. Patients were randomly divided into a training set (n=72) and a validation set (n=32). Risk factors for ICI were assessed using univariate and multivariate logistic regression analyses. A predictive nomogram was constructed based on the identified independent risk factors and evaluated for its performance.

**Results:**

Sixteen of the 104 patients (15.4%) developed a postoperative ICI. Multivariate logistic regression analysis identified a preoperative Karnofsky Performance Status (KPS) score ≤ 70 (OR = 16.55, 95% CI: 2.57–106.54, *P* = 0.003) and a drainage tube placement time ≥ 48 hours (OR = 15.42, 95% CI: 1.10–215.46, *P* = 0.042) as independent risk factors for ICI. A nomogram incorporating these two factors was developed and showed excellent discrimination, with an area under the curve (AUC) of 0.93 (95% CI: 0.87–0.99) in the training set and 0.92 (95% CI: 0.79–1.00) in the validation set. Calibration plots and decision curve analysis confirmed the nomogram’s accuracy and clinical utility.

**Conclusion:**

A low preoperative KPS score and prolonged drainage tube placement are significant independent predictors of ICI after glioblastoma surgery. The nomogram developed in this study provides a simple and accurate tool for risk stratification, which can assist clinicians in identifying high-risk patients and implementing targeted preventive measures.

## Introduction

According to the 2022 global cancer statistics released by the International Agency for Research on Cancer, malignant tumors of the brain and central nervous system (CNS) have become a significant global public health challenge. The data indicate that in 2022, malignant CNS tumors resulted in 248,305 deaths worldwide, accounting for 2.6% of all cancer-related deaths and ranking 12th most lethal malignancy (1). The 2021 World Health Organization (WHO) Classification of Tumors of the Central Nervous System establishes molecular features as a cornerstone of diagnosis. CNS WHO grade 4 tumors represent the most aggressive and malignant brain tumors, primarily including glioblastoma, Isocitrate Dehydrogenase (IDH)-wildtype, CNS WHO grade 4, and astrocytoma, IDH-mutant, CNS WHO grade 4 (2). In this paper, we will refer to these two tumor types collectively as high-grade gliomas (HGGs). For these tumors, the current standard of care involves maximal safe surgical resection followed by radiotherapy and adjuvant chemotherapy (3–5). While surgical intervention is crucial for cytoreduction, diagnosis, and relieving mass effect, it is not without significant risks. One of the most severe postoperative complications is intracranial infection (ICI), which can lead to meningitis, cerebritis, or abscess formation (6).

Postoperative ICI is associated with devastating consequences, including prolonged hospitalization, increased medical costs, severe neurological sequelae, and a significantly higher rate of morbidity and mortality (7, 8). The incidence of ICI after craniotomy for brain tumors has been reported to range from 1.4% to 9.5%, depending on the patient population and surgical complexity (9). For HGGs patients, who are often in a relatively immunocompromised state due to the disease itself and its treatments (such as steroids and chemotherapy), the risk of infection can be particularly high (10).

Although several studies have identified various potential risk factors for post-neurosurgical infections—such as prolonged surgery, cerebrospinal fluid (CSF) fistula, and the presence of foreign implants—most of these studies have focused on general neurosurgical populations (11–13). Specific research in HGGs cohorts has recently identified several important predictors. For instance, Nair et al. found that a low preoperative Karnofsky Performance Status (KPS) score, the use of a trapdoor scalp incision, and combined preoperative and postoperative steroid administration were independently associated with an elevated risk of SSI (14). Similarly, Scheer et al. noted trends suggesting that prior surgery and the implantation of foreign material might be influencing factors (15). However, the literature is not entirely consistent, and some studies, like the one by Hounchonou et al., focus more on the controversial impact of SSI on patient survival rather than on predictive factors (16). This highlights that while progress has been made, a lack of specific, reliable tools designed to predict the risk of ICI in the HGGs patient cohort persists. Early identification of high-risk patients would allow clinicians to implement targeted preventive measures and enhanced monitoring, thereby potentially reducing the incidence of this grave complication.

Nomograms are widely used statistical tools that provide a graphical representation of a prediction model, enabling clinicians to calculate the probability of a specific event for an individual patient. By integrating multiple significant variables, nomograms can offer a more personalized and accurate risk assessment compared to relying on single risk factors. Their user-friendly interface makes them a practical tool for clinical decision-making at the bedside (17).

Therefore, the aim of this study was to develop and validate a clinical nomogram to predict the probability of developing ICI following surgical resection for HGGs. By identifying key predictors from readily available patient and treatment data, we sought to create a practical tool to aid in risk stratification and guide clinical management in this vulnerable patient population.

## Methods

### Study participants

This retrospective study analyzed data from 108 patients who underwent HGGs surgery at our institution between January 2017 and August 2024. The inclusion criteria were as follows: (1) pathologically confirmed CNS WHO grade 4 high-grade glioma (including glioblastoma, IDH-wildtype, and astrocytoma, IDH-mutant); (2) underwent surgical resection of the tumor; (3) complete clinical, imaging, and follow-up data. The exclusion criteria were: (1) evidence of preoperative ICI; (2) concurrent severe systemic infection; (3) incomplete or missing medical records (n=4).

### Ethics approval

This study was approved by the Institutional Ethics Committee of Fujian Medical University Union Hospital, and the requirement for informed consent was waived due to the retrospective nature of the study (2025KY055).

### Data collection

Patient data was collected from the hospital’s electronic medical record system. We collected information on baseline characteristics, including age, sex, smoking and drinking history, history of diabetes, preoperative KPS score, and comorbidities such as organ dysfunction. Organ dysfunction was defined as the presence of pre-existing severe cardiac (New York Heart Association class III–IV), pulmonary (Global Initiative for Chronic Obstructive Lung Disease stage III–IV), hepatic (Child-Pugh class B or C), or renal (Chronic Kidney Disease stage 3–5) insufficiency. We also gathered clinicopathological and treatment-related data, including tumor recurrence, tumor diameter, whether the tumor invaded the ventricle, IDH 1 mutation status, history of chemoradiotherapy, use of artificial dura mater, total tumor resection status, duration of surgery, blood transfusion, preoperative and postoperative hypoproteinemia, duration of drainage tube placement, and incision CSF fistula. In this cohort, all patients had a prophylactic epidural closed, non-suction drain placed at the surgical site at the end of the operation. The timing of drain removal was not fixed but was based on the nature of the drainage fluid, typically removed when the fluid became clear or serosanguineous. We also collected data on perioperative corticosteroid use. According to our institution’s standard protocol for managing peritumoral edema, all patients in this cohort received a standardized dexamethasone regimen: an intravenous loading dose of 10 mg during anesthesia induction, followed by a maintenance dose of 4 mg every 6 hours (16 mg/day) postoperatively. Starting from the third postoperative day, if the patient’s condition was stable, the dose was tapered by half every 1–2 days until discontinuation, with a total course lasting 7–10 days. Most patients at our center received prophylactic antibiotics unless contraindicated (e.g., allergies or emergency surgery). This included an intravenous infusion of 1.5 g of cefuroxime within 60 minutes before skin incision. For surgeries lasting longer than 4 hours, an intraoperative dose was re-administered. All data were independently collected and cross-checked by two researchers to ensure accuracy. The final dataset was locked before statistical analysis.

### Definition of ICI

The diagnosis of postoperative ICI was based on a comprehensive assessment integrating clinical presentation, CSF analysis, and neuroimaging findings. A definitive diagnosis was established by a positive CSF culture. In the absence of a positive culture, a presumptive diagnosis of ICI (specifically meningitis in this cohort) was made according to established criteria (9, 18) if a patient exhibited clinical signs suggestive of meningitis (persistent fever >38.5°C accompanied by new-onset severe headache, nuchal rigidity, or altered mental status, after excluding other common postoperative causes) AND characteristic CSF abnormalities (pleocytosis [white blood cell count >10×10^6^/L], elevated protein [>0.45 g/L], and decreased glucose [<2.25 mmol/L]). Neuroimaging was performed in all suspected cases primarily to exclude non-infectious pathologies (e.g., hematoma) or contraindications to lumbar puncture (LP). Additionally, it provided supportive diagnostic evidence if cranial imaging revealed new or worsening findings, such as meningeal enhancement, cerebritis, or abscess formation. LP was performed selectively based on the aforementioned clinical suspicion, under strict aseptic conditions, and typically after imaging excluded significant mass effect. Supportive laboratory findings (e.g., peripheral leukocytosis) were also considered but were not primary diagnostic criteria.

### Statistical analysis

Statistical analyses were performed using SPSS version 27.0 (IBM Corp., Armonk, NY, USA) and R software version 4.1 (R Foundation for Statistical Computing, Vienna, Austria). The entire cohort of 104 eligible patients was randomly divided into a training set (n=72) and a validation set (n=32) at a 7:3 ratio.

Categorical variables were presented as numbers and percentages (n, %) and compared using the Chi-square test or Fisher’s exact test, as appropriate. Univariate logistic regression analysis was performed to identify potential risk factors associated with postoperative ICI. Variables with a p-value < 0.10 in the univariate analysis were included in the multivariate logistic regression analysis using a backward stepwise selection method to identify independent predictors. The results were reported as odds ratios (OR) with 95% confidence intervals (CI). A *P* -value < 0.05 was considered statistically significant.

A nomogram was constructed based on the final multivariate model to provide a visual tool for predicting the probability of ICI. The performance of the nomogram was assessed in both the training and validation sets. Its discriminative ability was evaluated using the area under the receiver operating characteristic (ROC) curve (AUC). The calibration of the nomogram was assessed using calibration plots and the Hosmer-Lemeshow goodness-of-fit test. Finally, decision curve analysis (DCA) was conducted to evaluate the clinical utility and net benefit of the nomogram across a range of threshold probabilities.

## Results

### Patient characteristics and univariate analysis

**Figure 1:**
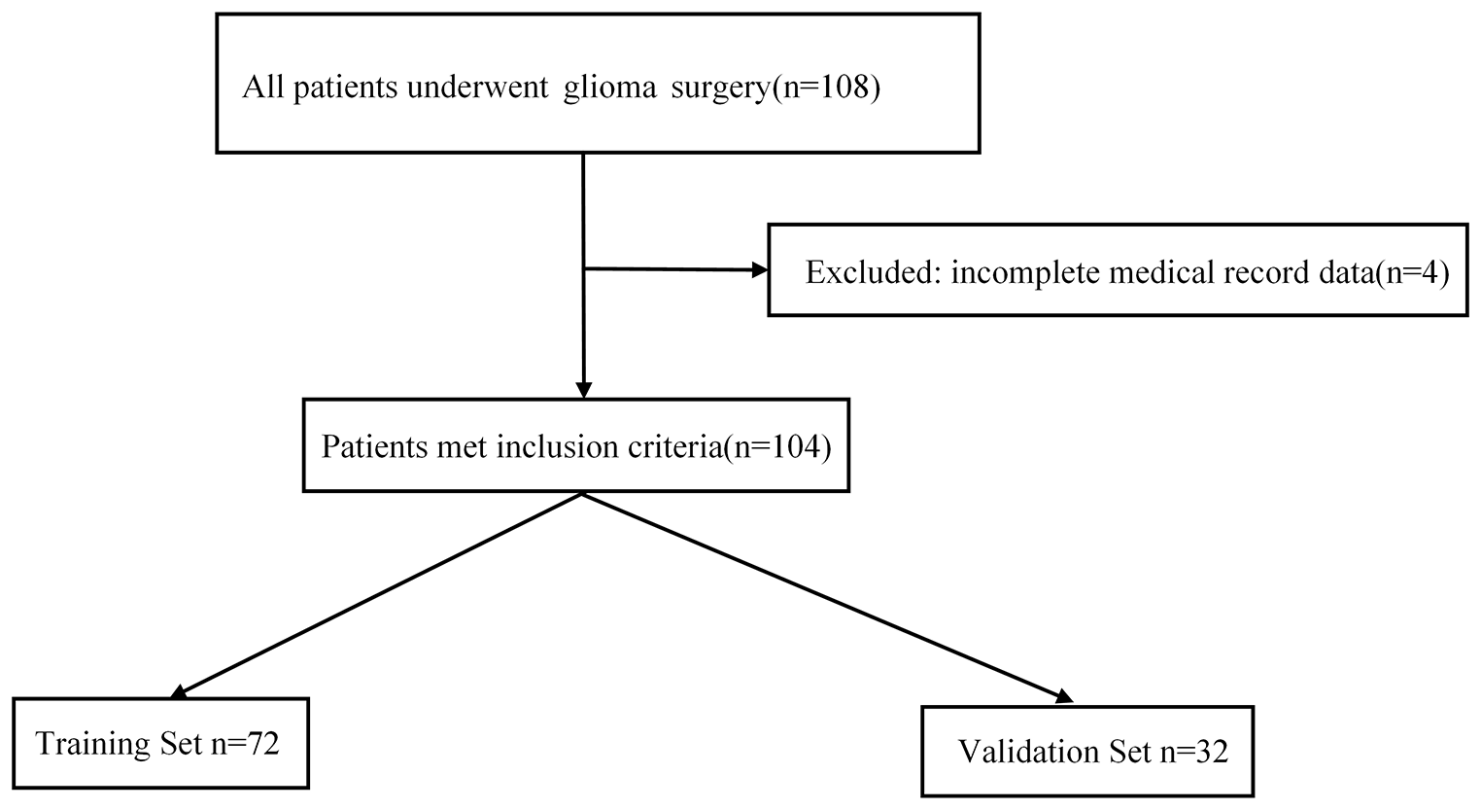
Flowchart of patient selection for the study.

A total of 108 patients who underwent surgery for HGGs were initially screened for this study. After excluding 4 patients with incomplete medical records, 104 patients were ultimately included in the analysis (see **Figure 1** for the screening flowchart). Within the cohort, postoperative ICI occurred in 16 patients (15.4%), with a median time from surgery to diagnosis of 7 days (range: 3–11 days). All infection cases were diagnosed before the initiation of adjuvant chemoradiotherapy. Based on the diagnostic criteria described in the Methods, all 16 ICI patients were diagnosed with postoperative meningitis. Of these, 2 cases (12.5%) were culture-positive (definitive diagnosis), while 14 (87.5%) met the criteria for presumptive meningitis. Detailed clinical and diagnostic data for each of these 16 cases are presented in **Supplementary Table S1**. All 16 patients underwent cranial imaging prior to lumbar puncture. This imaging confirmed the absence of mass effect, hemorrhage, or hydrocephalus in all cases, establishing the safety profile for the LP. Furthermore, these scans showed no definitive radiological signs of progressed infection, such as meningeal enhancement, cerebritis, or abscess formation. This lack of radiological findings is consistent with an early-stage meningitis diagnosis, which is often radiologically occult, and confirms the diagnosis was established based on the clinical and CSF findings. Fortunately, all infected patients were effectively managed with timely targeted antibiotic therapy, and none required a second surgical intervention such as debridement or abscess drainage. The final cohort was randomly assigned to a training set (n=72) and a validation set fg(n=32).

The results of the univariate analysis for potential risk factors of ICI are shown in **Table 1**. The analysis revealed that preoperative KPS score (≤ 70), chemoradiotherapy, organ dysfunction, tumor diameter (≥ 5cm), tumor invasion of the ventricle, artificial dura mater implantation, non-total tumor resection, prolonged drainage tube placement time (≥ 48h), and IDH 1 mutations were significantly associated with an increased risk of postoperative ICI (*P* < 0.05). The variable assignments are shown in **Table 2**.

**Table 1:** Univariate analysis results of the ICI following HGGs surgery.

Variables	Total (n = 104)	No infection group (n = 88)	Infection group (n = 16)	Statistic	*P*
Age, n(%)				χ²=0.63	0.426
< 60y	69 (66.35)	57 (64.77)	12 (75.00)		
≥ 60y	35 (33.65)	31 (35.23)	4 (25.00)		
Sex, n(%)				χ²=0.02	0.894
Female	70 (67.31)	59 (67.05)	11 (68.75)		
Male	34 (32.69)	29 (32.95)	5 (31.25)		
Recurrence, n(%)				χ²=0.70	0.404
No	77 (74.04)	67 (76.14)	10 (62.50)		
Yes	27 (25.96)	21 (23.86)	6 (37.50)		
Smoking, n(%)				χ²=1.53	0.217
No	75 (72.12)	66 (75.00)	9 (56.25)		
Yes	29 (27.88)	22 (25.00)	7 (43.75)		
Drinking, n(%)				χ²=2.26	0.133
No	69 (66.35)	61 (69.32)	8 (50.00)		
Yes	35 (33.65)	27 (30.68)	8 (50.00)		
Diabetes, n(%)				χ²=1.561	0.212
No	98 (94.23)	84(95.45)	14 (87.50)		
Yes	6 (5.27)	4 (4.55)	2 (12.50)		
Preoperative KPS score, n(%)				χ²=20.69	<.001
> 70 points	88 (84.62)	81 (92.05)	7 (43.75)		
≤ 70 points	16 (15.38)	7 (7.95)	9 (56.25)		
Chemoradiotherapy, n(%)				χ²=5.24	0.022
No	88 (84.62)	78 (88.64)	10 (62.50)		
Yes	16 (15.38)	10 (11.36)	6 (37.50)		
Organ dysfunction, n(%)				-	0.025
No	99 (95.19)	86 (97.73)	13 (81.25)		
Yes	5 (4.81)	2 (2.27)	3 (18.75)		
Tumor diameter, n(%)				χ²=4.03	0.045
< 5cm	50 (48.08)	46 (52.27)	4 (25.00)		
≥ 5cm	54 (51.92)	42 (47.73)	12 (75.00)		
Tumor invades ventricle, n(%)				χ²=6.60	0.010
No	76 (73.08)	69 (78.41)	7 (43.75)		
Yes	28 (26.92)	19 (21.59)	9 (56.25)		
Preoperative hypoproteinemia, n(%)				χ²=0.007	0.934
No	97 (93.27)	82 (93.18)	15 (93.75)		
Yes	7 (6.73)	6 (6.82)	1 (6.25)		
Emergency surgery, n(%)				χ²=3.27	0.071
No	94 (90.38)	82 (93.18)	12 (75.00)		
Yes	10 (9.62)	6 (6.82)	4 (25.00)		
Duration of operation, n(%)				χ²=2.94	0.086
< 4h	53 (50.96)	48 (54.55)	5 (31.25)		
≥ 4h	51 (49.04)	40 (45.45)	11 (68.75)		
Artificial dura mater implantation, n(%)				χ²=7.48	0.006
No	38 (36.54)	37 (42.05)	1 (6.25)		
Yes	66 (63.46)	51 (57.95)	15 (93.75)		
Blood transfusion, n(%)				χ²=1.98	0.159
No	92 (88.46)	80 (90.91)	12 (75.00)		
Yes	12 (11.54)	8 (9.09)	4 (25.00)		
Total tumor resection, n(%)				χ²=4.91	0.027
No	31 (29.81)	22 (25.00)	9 (56.25)		
Yes	73 (70.19)	66 (75.00)	7 (43.75)		
Drainage tube placement time, n(%)				χ²=18.96	<.001
< 48h	96 (92.31)	86 (97.73)	10 (62.50)		
≥ 48h	8 (7.69)	2 (2.27)	6 (37.50)		
Postoperative hypoproteinemia, n(%)				χ²=0.26	0.613
No	45 (43.27)	39 (44.32)	6 (37.50)		
Yes	59 (56.73)	49 (55.68)	10 (62.50)		
Incision cerebrospinal fluid fistula, n(%)				–	0.111
No	100 (96.15)	86 (97.73)	14 (87.50)		
Yes	4 (3.85)	2 (2.27)	2 (12.50)		
Preoperative prophylactic use of antibiotics, n(%)				χ²=0.31	0.578
No	12 (11.54)	9 (10.23)	3 (18.75)		
Yes	92 (88.46)	79 (89.77)	13 (81.25)		
Corticosteroid Duration, n(%)				χ²=0.04	0.841
≤ 7 days	55 (52.88)	46 (52.27)	9 (56.25)		
> 7 days	49 (47.12)	42 (47.73)	7 (43.75)		
IDH 1 Mutations, n(%)				χ²=6.73	0.009
No	81 (77.88)	73 (82.95)	8 (50.00)		
Yes	23 (22.12)	15 (17.05)	8 (50.00)		

ICI, Intracranial infection; HGGs, high-grade gliomas; KPS score, Karnofsky Performance Status Scale; IDH 1 Mutations, Isocitrate Dehydrogenase 1 Mutations; χ², Chi-square test; -, Fisher exact. Bold values indicate P < 0.05.

**Table 2:** Quantification and assignment for risk factors of ICI after HGGs surgery.

Factor	Variables	Quantification and assignment
Age	X1	"0" for < 60y, "1" for ≥ 60y
Sex	X2	"0" for Female, "1" for Male
Recurrence	X3	"0" for No, "1" for Yes
Smoking	X4	"0" for No, "1" for Yes
Drinking	X5	"0" for No, "1" for Yes
Diabetes	X6	"0" for No, "1" for Yes
preoperative KPS score	X7	"0" for > 70 points, "1" for ≤ 70 points
Chemoradiotherapy	X8	"0" for No, "1" for Yes
Organ dysfunction	X9	"0" for No, "1" for Yes
Tumor diameter	X10	"0" for < 5cm, "1" for ≥ 5cm
Tumor invades ventricle	X11	"0" for No, "1" for Yes
Preoperative hypoproteinemia	X12	"0" for No, "1" for Yes
Emergency surgery	X13	"0" for No, "1" for Yes
Duration of operation	X14	"0" for < 4h, "1" for ≥ 4h
Artificial dura mater implantation	X15	"0" for No, "1" for Yes
Blood transfusion	X16	"0" for No, "1" for Yes
Total tumor resection	X17	"0" for Yes, "1" for No
Drainage tube placement time	X18	"0" for < 48h, "1" for ≥ 48h
Postoperative hypoproteinemia	X19	"0" for No, "1" for Yes
Incision cerebrospinal fluid fistula	X20	"0" for No, "1" for Yes
Preoperative prophylactic use of antibiotics	X21	"0" for No, "1" for Yes
Corticosteroid Duration	X22	"0" for ≤ 7 days, "1" for> 7 days
IDH 1 Mutations	X23	"0" for No, "1" for Yes
Intracranial infection	Y	"0" for No, "1" for Yes

ICI, Intracranial infection; HGGs, high-grade gliomas; KPS score, Karnofsky Performance Status Scale; IDH 1 Mutations, Isocitrate Dehydrogenase 1 Mutations.

### Multivariate analysis of independent risk factors

Variables that were statistically significant in the univariate analysis were included in the multivariate logistic regression model. The results (**Table 3**) identified preoperative KPS score (OR = 16.55, 95% CI: 2.57–106.54, *P* = 0.003) and drainage tube placement time (OR = 15.42, 95% CI: 1.10–215.46, *P* = 0.042) as independent risk factors for developing ICI after HGGs surgery. Although tumor invasion of the ventricle showed a strong trend, it did not reach statistical significance in the multivariate model (OR = 6.21, 95% CI: 0.94-41.15, *P* = 0.058).

**Table 3:** Multivariate logistic regression analysis for ICI after HGGs surgery.

Variables	β	S.E	Z	*P*	OR (95%CI)
Preoperative KPS score	2.81	0.95	2.95	**0.003**	16.55 (2.57 ~ 106.54)
Organ dysfunction	2.34	2.04	1.15	0.251	10.38 (0.19 ~ 564.85)
Tumor invades ventricle	1.83	0.96	1.89	0.058	6.21 (0.94 ~ 41.15)
Drainage tube placement time	2.74	1.35	2.03	**0.042**	15.42 (1.10 ~ 215.46)
IDH 1 Mutations	0.45	1.05	0.43	0.669	1.57 (0.20 ~ 12.22)

ICI, Intracranial infection; HGGs, high-grade gliomas; KPS score, Karnofsky Performance Status Scale; IDH 1 Mutations, Isocitrate Dehydrogenase 1 Mutations; β, regression coefficient; OR, Odds Ratio; CI, Confidence Interval. Bold values indicate P < 0.05.

### Nomogram construction and performance

**Figure 2:**
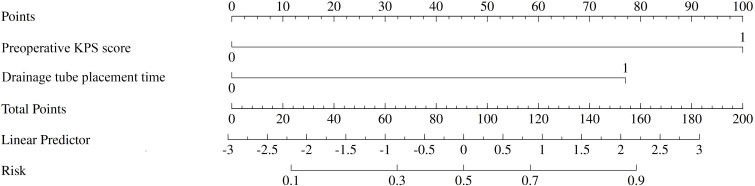
Nomogram for predicting the risk of postoperative intracranial infection (ICI) after high-grade gliomas (HGGs) surgery. To use the nomogram, locate the patient’s values on the ‘preoperative KPS score’ and ‘Drainage tube placement time’ axes. Draw a vertical line up to the ‘Points’ axis to determine the score for each variable. Sum the scores to get the ‘Total Points’ and draw a vertical line down to the ‘Risk’ axis to find the patient’s probability of developing ICI.

A predictive nomogram was constructed to provide a visual, user-friendly tool for risk assessment, incorporating the two independent risk factors identified through multivariate logistic regression (**Figure 2**). In this model, each predictor is assigned a point value based on its regression coefficient. The sum of these points corresponds to a total score, which is then mapped to the estimated probability of developing a postoperative ICI.

**Figure 3:**
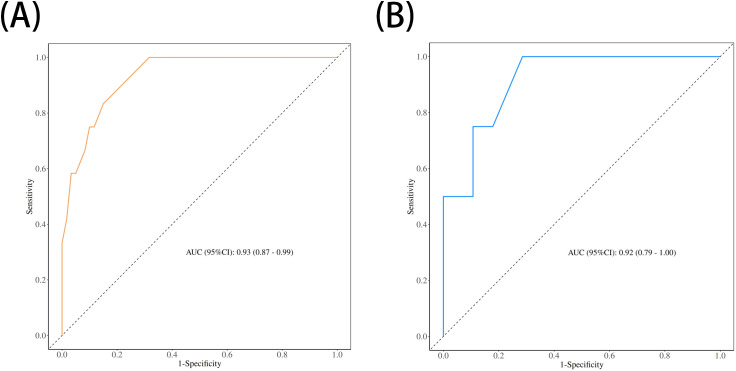
Receiver operating characteristic (ROC) curves for the nomogram's performance. The area under the curve (AUC) demonstrates the model's discriminative ability in **(A)** the training set (AUC = 0.93) and **(B)** the validation set (AUC = 0.92).

**Figure 4:**
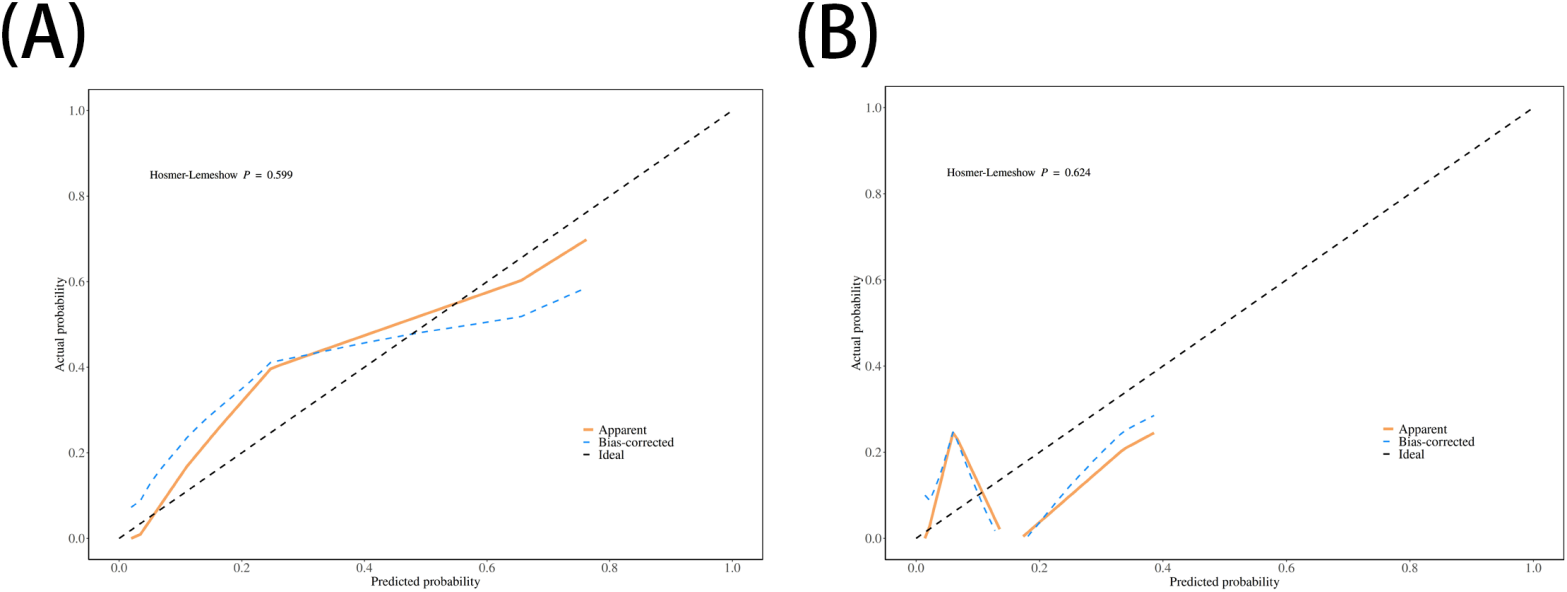
Calibration plots for the nomogram. The plots compare the nomogram-predicted probabilities of ICI (x-axis) with the actual observed ICI rates (y-axis) for **(A)** the training set and **(B)** the validation set. The dashed line represents an ideal model, and the closeness of the solid line to the dashed line indicates good calibration.

**Figure 5:**
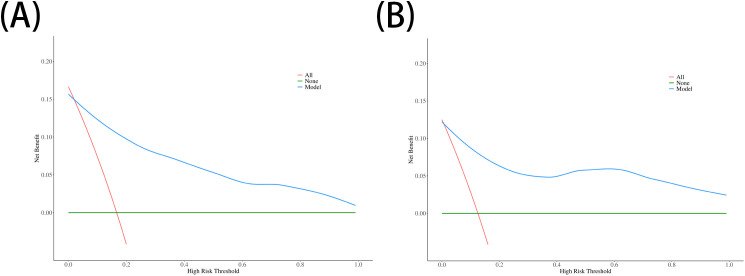
Decision curve analysis (DCA) for the nomogram. The y-axis represents the net benefit. The plots for **(A)** the training set and **(B)** the validation set show that using the nomogram (blue line) to make clinical decisions offers a greater net benefit across a wide range of threshold probabilities compared to treating all patients (red line) or treating no patients (green line).

The performance of the nomogram was systematically evaluated for its discrimination, calibration, and clinical utility. The model’s discriminative ability, assessed by the AUC, was found to be excellent. The nomogram achieved an AUC of 0.93 (95% CI: 0.87–0.99) in the training cohort (**Figure 3A**) and demonstrated similarly strong performance in the validation cohort with an AUC of 0.92 (95% CI: 0.79–1.00) (**Figure 3B**). To assess calibration, calibration plots were generated, which showed strong concordance between the nomogram-predicted probabilities and the actual observed infection rates for both the training (**Figure 4A**) and validation (**Figure 4B**) sets. This was further supported by the Hosmer-Lemeshow goodness-of-fit test, which yielded non-significant results (*P* = 0.599 and *P* = 0.624, respectively), indicating the model is well-calibrated. Finally, the clinical utility of the nomogram was confirmed through Decision Curve Analysis (DCA). The DCA plots (**Figure 5**) demonstrated that utilizing the nomogram for clinical decision-making provides a superior net benefit across a wide spectrum of threshold probabilities compared to strategies of treating all or no patients.

## Discussion

ICI following HGGs surgery is a serious complication that significantly worsens patient prognosis. The infection rate in our cohort was 15.4%, which is higher than the commonly reported rates for general craniotomies, underscoring the particular vulnerability of the HGGs patient population. We believe this higher infection rate reflects the success of our clinical practice in achieving early diagnosis and timely intervention through close monitoring. The fact that all diagnosed infections were effectively controlled with antibiotic therapy before progressing to a severe stage requiring surgical intervention (such as brain abscess) validates the accuracy of our diagnoses and the effectiveness of our clinical management. Therefore, identifying and intervening on these early infection risks is crucial for improving patient outcomes. In this study, we analyzed a range of clinical factors and developed a simple yet effective nomogram for predicting postoperative ICI in HGGs patients. Our multivariate analysis conclusively identified a low preoperative KPS score (≤ 70) and prolonged drainage tube placement (≥ 48h) as powerful independent risk factors for infection.

A low preoperative KPS score is a robust indicator of a patient’s poor overall health status. This condition often correlates with malnutrition, sarcopenia, and a compromised immune system, which may be exacerbated by the tumor itself or by prior treatments like corticosteroid therapy (19). Patients with poor functional status have diminished physiological reserves to withstand the significant stress of major surgery and are inherently more susceptible to opportunistic pathogens. A weakened immune response can impair all phases of wound healing, from hemostasis to tissue remodeling, creating a favorable environment for bacterial invasion and proliferation at the surgical site (20). This finding aligns with broader surgical literature indicating that poor baseline health is a key determinant of postoperative complications, including infections (21). Crucially, our result is strongly supported by the recent large-scale study by Nair et al., who also identified a lower preoperative KPS as an independent predictor of SSI in a cohort of over 900 glioblastoma resections. This consistency across studies solidifies the KPS score as a reliable and critical factor for risk stratification in this patient population (14). Therefore, preoperative optimization, including nutritional support and physical rehabilitation for patients with low preoperative KPS scores, could be a critical strategy to mitigate infection risk.

Prolonged epidural drain placement (≥48 hours) was the second independent risk factor identified in our study, a finding highly consistent with numerous studies in the neurosurgical field (22–24). The mechanisms by which drains increase infection risk are multifactorial: first, as an invasive device, it breaches the skin’s physical barrier, providing a direct conduit for external flora to ascend into the sterile surgical field; second, the drain acts as a foreign body, which can induce a local inflammatory response and provide a surface for bacterial biofilm formation, wherein bacteria are often resistant to systemic antibiotics (25). Despite these risks, the placement of prophylactic drains remains a key measure in clinical practice to prevent postoperative hematomas and fluid collections. At our center, all patients routinely have an epidural drain placed. This strategy is based on a careful risk-benefit analysis and surgeon preference, specifically choosing epidural over subdural drainage to leverage the natural barrier of the dura mater, thereby avoiding direct contact between the drain and the resection cavity and cortical vessels, which reduces the risk of iatrogenic subdural hemorrhage upon removal (26). Furthermore, our criteria for removal are not based on a fixed schedule but on clinical judgment—specifically, continuing drainage until the bloody CSF becomes clear, with the aim of reducing its irritant effect on the meninges to stabilize intracranial pressure and patient temperature. However, our data clearly reveal a critical clinical dilemma: while our drainage strategy is designed to mitigate hemorrhagic complications, the duration of drain placement itself constitutes a powerful, independent risk factor for infection. Our analysis provides an important clinical reference point for this dilemma—the 48-hour threshold. This finding strongly advocates for a more standardized, protocol-driven approach to drain management, emphasizing not only strict aseptic techniques but also mandating the earliest possible removal of the drain (ideally within 48 hours) when clinically permissible, to achieve an optimal balance between preventing hematomas and controlling infection risk.

It is noteworthy that several other factors were significant in the univariate analysis but did not retain their independent predictive value in the final multivariate model. These include a history of chemoradiotherapy, organ dysfunction, large tumor diameter (≥5cm), tumor invasion of the ventricle, use of artificial dura mater, non-total tumor resection, and IDH 1 mutation status. The loss of significance in the multivariate analysis may be attributable to the limited sample size and the number of infection events in our cohort, or potential confounding effects between variables. However, their clinical relevance should not be overlooked. For example, prior chemoradiotherapy can lead to myelosuppression and impaired tissue healing (27). Large tumors often require more extensive resection and longer operative times, while invasion into the ventricular system can disrupt CSF dynamics and the ependymal barrier, potentially facilitating the dissemination of pathogens (28). Similarly, artificial dura mater, as a foreign implant, can trigger inflammatory responses and serve as a nidus for infection (29), and subtotal resection may leave behind a necrotic tumor bed susceptible to infection, as necrotic tissue can provide ample nutrients for microbial proliferation (30). The association of IDH1 mutation with an increased risk of infection in the univariate analysis of this study is a noteworthy finding. The potential mechanism may be related to the profound changes induced by IDH mutations in the tumor microenvironment. Specifically, the production of the oncometabolite 2-hydroxyglutarate (2-HG) is known to disrupt immune cell function (31). Furthermore, IDH-mutant tumors often form a unique hypoxic microenvironment, which can amplify immunosuppressive effects by activating pathways such as HIF-1α, thereby enhancing lactate production and adenosine signaling (32). Collectively, these biological features may lead to impaired local immune function, rendering patients more susceptible to postoperative infections, although it did not constitute an independent predictor in our multivariate model. It is worth noting that the duration of surgery, a recognized risk factor for central nervous system infection after craniotomy, warrants in-depth discussion. Numerous studies have shown a positive correlation between surgical duration and infection rates, often considering surgery lasting over four hours as a threshold for a significant increase in risk. The mechanism is generally attributed to prolonged exposure of the surgical area to potential contaminants, increased local tissue trauma, and the immunosuppressive effects of prolonged anesthesia (33). In our cohort, prolonged surgery (≥4 hours) also showed a trend toward significance in the univariate analysis (p=0.086) and was thus included in the multivariate model. However, it ultimately did not emerge as an independent predictor. We believe this may be due to its predictive effect being confounded by other variables in the final model. Longer surgical times are often associated with larger tumor volumes and greater surgical complexity, which in turn may be intrinsically linked to patients’ lower preoperative KPS scores. Therefore, although surgical duration is undoubtedly an important component of the overall infection risk, its predictive weight may have been captured by the more dominant independent predictors in our model, such as the preoperative KPS score. These factors likely still contribute to the overall risk profile and warrant further investigation in larger, multicenter studies to clarify their roles.

The nomogram developed from the two independent predictors demonstrated excellent performance. With AUC values of 0.93 and 0.92 in the training and validation cohorts, respectively, the model shows outstanding discriminative ability. The calibration plots and DCA further confirmed the model’s reliability and clinical utility. The strength of this nomogram lies in its simplicity and practicality. Based on two easily accessible clinical parameters, it can be conveniently used at the bedside to stratify patients into different risk categories. For patients identified as high-risk, clinicians can proactively implement enhanced preventive strategies. For example, a patient with a preoperative KPS score of 60 who is anticipated to have a difficult surgery with a potential drainage time exceeding 48 hours would be flagged as high-risk. This high-risk designation could trigger a series of specific management interventions, including: (1) consulting with a nutrition specialist for preoperative optimization; (2) employing stricter aseptic techniques during drain management; (3) developing a protocol for the earliest possible drain removal; (4) considering extended or broad-spectrum antibiotic prophylaxis; and (5) intensifying monitoring for early clinical signs of infection and lowering the threshold for performing imaging or CSF analysis. This standardized risk assessment moves beyond general risk awareness to an actionable, patient-specific treatment pathway.

## Limitations

This study has several limitations that should be considered when interpreting its findings. First, the inherent limitations of the study design stem from its retrospective and single-center nature, which may restrict the generalizability of the results to different healthcare settings or patient populations. Second, the statistical power of the study is limited. Our sample size is relatively small, with only 16 infection events, which has resulted in wide confidence intervals for the odds ratios in the multivariate analysis. More importantly, the limited sample size may be insufficient to detect modest but clinically significant effects of other potential risk factors. Therefore, this study should be regarded as a hypothesis-generating exploratory analysis, and its conclusions urgently require validation by larger-scale, prospective, multicenter studies. Third, on a methodological level, the seven-year span of the study (2017–2024) covers two updates to the WHO classification of CNS tumors. To maintain statistical power and avoid potential bias, we combined CNS WHO grade 4 tumors with similar clinical behavior and perioperative management strategies for analysis. While this approach is reasonable for studying a common complication like postoperative infection, it may mask potential differences between molecular subtypes. Finally, regarding model performance and validation, although our nomogram demonstrated excellent discriminative ability in internal validation (AUC > 0.9), we acknowledge that these performance metrics may be overestimated due to the single-center design and limited sample size. The model has not yet been validated in an independent external cohort; therefore, its robustness and reliability await further confirmation before wider clinical application, and the current results should be interpreted with caution.

## Conclusion

This study successfully developed and validated a novel nomogram to predict postoperative ICI in patients with HGGs. This practical tool, based on two readily available clinical variables (preoperative KPS score and drainage tube placement time)—demonstrated excellent predictive accuracy and calibration. The nomogram’s primary clinical application lies in its ability to stratify patients, enabling clinicians to identify high-risk individuals who may benefit from enhanced surveillance, targeted prophylactic measures, and more informed patient counseling. Future prospective, multicenter studies with larger cohorts are essential to externally validate these findings, further refine the prediction model, and ultimately improve surgical outcomes for this vulnerable patient population.

## Data Availability

The original contributions presented in the study are included in the article/**Supplementary Material**. Further inquiries can be directed to the corresponding authors.
