# Supplementary material for: Risk stratification for intracranial infection after high-grade gliomas surgery: a nomogram development and validation study
Source: Front Oncol. 2025 Nov 19;15:1697966. doi: 10.3389/fonc.2025.1697966 (PMC12672262; doi:10.3389/fonc.2025.1697966)
Supplement: Supplementary file 1 [file Table1.docx]

| **Supplementary Table S1.** Detailed Clinical and Diagnostic Findings for 16 Patients with Postoperative Intracranial Infection | | | | | | |
| --- | --- | --- | --- | --- | --- | --- |
| Case ID | Days to Diagnosis | Clinical Signs | CSF WBC (10^6^/L) | CSF Protein (g/L) | CSF Glucose (mmol/L) | CSF Culture |
| 1 | 3 | Fever, Headache, Neck Stiffness | 1666 | 2.24 (H) | 1.79 (L) | Negative |
| 2 | 8 | Fever, Altered Consciousness | 401 | 2.12 (H) | 2.01 (L) | Negative |
| 3 | 7 | Fever, Severe Headache | 1800 | 1.47 (H) | 1.81 (L) | Negative |
| 4 | 3 | Fever, Neck Stiffness | 17029 | 2.11 (H) | 1.47 (L) | Negative |
| 5 | 3 | Fever, Headache | 2701 | 2.34 (H) | 1.66 (L) | Negative |
| 6 | 7 | Fever, Headache, Neck Stiffness | 1626 | 0.77 (H) | 2.09 (L) | Klebsiella pneumoniae |
| 7 | 11 | Fever, Altered Consciousness | 237 | 0.96 (H) | 2.14 (L) | Negative |
| 8 | 3 | Fever, Severe Headache | 1193 | 1.04 (H) | 1.20 (L) | Negative |
| 9 | 7 | Fever, Neck Stiffness | 388 | 1.76 (H) | 2.02 (L) | Negative |
| 10 | 6 | Fever, Headache | 1879 | 1.85 (H) | 1.25 (L) | Staphylococcus epidermidis |
| 11 | 9 | Fever, Headache, Neck Stiffness | 407 | 0.99 (H) | 2.04 (L) | Negative |
| 12 | 3 | Fever, Altered Consciousness | 12017 | 3.71 (H) | 0.98 (L) | Negative |
| 13 | 10 | Fever, Severe Headache | 389 | 0.91 (H) | 2.13 (L) | Negative |
| 14 | 7 | Fever, Neck Stiffness | 678 | 0.94 (H) | 1.94 (L) | Negative |
| 15 | 8 | Fever, Headache | 557 | 0.91 (H) | 2.10 (L) | Negative |
| 16 | 3 | Fever, Headache, Neck Stiffness | 3360 | 1.75 (H) | 1.14 (L) | Negative |

**(L):** Low (below diagnostic threshold of <2.25 mmol/L); **(H):** High (above diagnostic threshold of >0.45 g/L).

**CSF:** Cerebrospinal Fluid, **WBC:** White Blood Cell
